# The Validation Study of the Stress and Anxiety to Viral Epidemics−6 Scale Among Patients With Cancer in the COVID-19 Pandemic

**DOI:** 10.3389/fpsyt.2022.811083

**Published:** 2022-04-14

**Authors:** Hyeyeong Kim, Harin Kim, Hyuk Joo Lee, Eulah Cho, Su-Jin Koh, Oli Ahmed, Seockhoon Chung

**Affiliations:** ^1^Department of Hematology and Oncology, Ulsan University Hospital, University of Ulsan College of Medicine, Seoul, South Korea; ^2^Department of Psychiatry, Asan Medical Center, University of Ulsan College of Medicine, Seoul, South Korea; ^3^Department of Public Medical Service, Seoul National University Bundang Hospital, Seongnam, South Korea; ^4^Department of Psychiatry, Seoul Medical Center, Seoul, South Korea; ^5^Department of Psychology, University of Chittagong, Chattogram, Bangladesh; ^6^National Centre for Epidemiology and Population Health, Australian National University, Canberra, ACT, Australia

**Keywords:** COVID-19, cancer, anxiety, stress, pandemic

## Abstract

**Introduction:**

The study aimed to explore the psychometric properties of the Stress and Anxiety to Viral Epidemics-6 (SAVE-6) scale among patients with cancer who are in serious situations in the current COVID-19 pandemic.

**Methods:**

The survey included questions on the participants' demographic information, clinical history of cancer (including cancer type, stage, current treatment or diagnosis of complete remission), and scores on rating scales, including the SAVE-6 scale, Coronavirus Anxiety Scale (CAS), and the Patient Health Questionnaire-9 (PHQ-9).

**Results:**

The confirmatory factor analysis (CFA) results determined that the model fits the single factor structure of the SAVE-6 scale among patients with cancer. The multi-group CFA showed that SAVE-6 can measure the anxiety response in a similar way across multiple variables, such as sex, presence of clinical depression, being in a state of complete remission, or currently undergoing cancer treatment. The SAVE-6 scale showed good reliability (Cronbach's alpha = 0.819) and convergent validity with the rating scales, such as CAS [r = 0.348 (95% CI, 0.273–0.419), *p* < 0.001] and PHQ-9 items score [r = 0.251 (95% CI, 0.172–0.328), *p* < 0.001].

**Conclusions:**

This study confirms SAVE-6 as a reliable and valid rating scale for measuring the anxiety response of patients with cancer during the current COVID-19 pandemic.

## Introduction

In the era of the COVID-19 pandemic, cancer care is rapidly changing due to the chronic shortage of medical personnel, hospital beds, and personal protective equipment, including masks, gowns, and gloves. Moreover, both patients and medical staff might maintain social distancing as a preventive measure for viral infections. For patients with cancer, the direct consequence of treatment delay or non-adherence is a delayed diagnosis or stage shift, against which clinicians take utmost precautions (1). As cancer treatment deteriorates and the benefit of cancer therapy remains the same in the era of COVID-19 pandemic, various predicaments have recently emerged among patients, such as higher rates of psychiatric problems, including depression, anxiety, post-traumatic stress disorder, insomnia, and impulsivity (2). The main strategies for cancer treatment should include the prevention of COVID-19. The risk and benefit for active intervention in the cancer population must be individually considered. Treatment modalities, such as chemotherapy and elective surgery, could be delayed if patients have a low risk of disease progression. Minimizing outpatient visit numbers via telemedicine may further prevent potential viral exposure (3). Recent articles suggest that parsimonious radiation therapy as a short course radiation therapy could be a reasonable clinical strategy to alleviate considerable clinical burden and protect cancer patients at risk of viral infection (4, 5). According to the Society of Surgical Oncology, clinicians are required to triage their cancer patients by medical urgency and defer surgery accordingly. Although there are some differences among the cancer types and stages, cancer surgery should be deferred for at least 3 months or more, if possible (6). For instance, maximizing neoadjuvant chemotherapy is recommended as an alternative to limiting surgical procedures (7).

Patients with cancer have a higher level of psychological distress throughout the disease trajectory (8, 9) and are at a higher risk of major psychiatric disorders, including suicide, than the general population (10, 11). Considering that the majority of cancer patients experience significant distress from either physical or psychosocial difficulties (12), it would be reasonable to regard these patients as vulnerable to mental health problems. Preceding research indicated that ~25% of patients with cancer reported anxiety symptoms during the COVID-19 pandemic era (2). Another study revealed that a higher level of distress from the COVID-19 pandemic correlated with a higher level of anxiety and fear in patients with cancer (13). Moreover, the distress stemmed from the pandemic itself, as well as from the interruptions in cancer care services (14), which could delay the diagnosis or proper management of cancer. Indeed, the fear and anxiety of contracting COVID-19 led to a greater risk of the postponement of scheduled chemotherapy (15), and the refusal of cancer-related procedure and surgery (16). Anxiety symptoms related to cancer diagnosis, treatments, or prognosis should be assessed separately from the anxiety arising from the pandemic. The pre-existing rating scales for anxiety symptoms, such as Generalized Anxiety Disorders-7 items (17), State and Trait Anxiety Inventory (18), or Hamilton Anxiety Rating Scale (19), might be insufficient for measuring patients' anxiety symptoms, specifically those elicited in response to the COVID-19 pandemic. To measure the virus-related anxiety of patients with cancer, viral-specific anxiety rating scales are needed.

We developed the Stress and Anxiety to Viral Epidemics-6 items (SAVE-6) scale for measuring a patient's anxiety response specifically to viral epidemics (20). The SAVE-6 is derived from the SAVE-9 scale, which was developed for measuring the work-related stress and anxiety response of healthcare workers to the COVID-19 outbreak (21). Nine items of the SAVE-9 scale were clustered into two factors: factor I—anxiety about the epidemic (items 1, 2, 3, 4, 5, and 8) and factor II—work-related stress associated with the epidemic (items 6, 7, and 9). Factor I included the virus-related anxiety component, and its application was validated among the general population (20). Factor II included the work-related stress components, and its application for measuring COVID-19 associated work-related stress was validated among healthcare workers (22). The SAVE-6 scale was validated among the general population using samples in Korea (20), Lebanon (23), and the United States (24). Additionally, we applied this scale to a special population, including medical students (25) or public workers (26). In this study, we aimed to explore the psychometric properties of the SAVE-6 scale among cancer patients in serious situations during the COVID-19 pandemic.

## Methods

### Patients and Study Design

This study was conducted between December 7, 2020, and February 9, 2021, among patients with cancer who visited the Ulsan University Hospital, Ulsan, Korea. A paper-survey form was given to the patients who provided informed consent for their participation. They voluntarily responded to this survey, and an e-gift coupon valued at approximately 5 dollars was provided for their participation. The study protocol was approved by the Institutional Review Board of the Ulsan University Hospital (2020-1055). The survey form included questions on the participant's age, sex, marital status, educational level, religion, occupation, current alcohol or tobacco use, and past psychiatric illnesses. The cancer types, cancer stages, current cancer treatment, or diagnosis of the complete remission were also gathered from the participants.

### Rating Scales

#### Stress and Anxiety to Viral Epidemics-6

The SAVE-6 is a self-report rating scale, which was developed for measuring the anxiety response of individuals to viral epidemics (20). It was derived from the factor I of the SAVE-9 scale, which was developed for measuring healthcare workers' work-related stress and anxiety response to viral epidemics (21). Each of the 6 items can be rated on a five-point Likert scale ranging from 0 (never) to 4 (always). A high score reflects a severe degree of anxiety response to the viral epidemic. The appropriate cut off was reported as point 15 in accordance with a mild degree of generalized anxiety (Generalized Anxiety Disorder-7 items scale score ≥ 5) among the general population (20).

#### Coronavirus Anxiety Scale

The CAS is a brief self-reported screening tool for “coronaphobia,” clinical anxiety, and fear associated with the COVID-19 crisis (27). The five items, which measure dizziness, sleep disturbance, tonic immobility, appetite loss, and abdominal distress, can be rated on a five-point scale from 0 (not at all) to 4 (nearly every day). In this study, we applied the Korean version of the CAS scale, and the psychometric properties of the CAS have been validated in South Korea (28). In this sample, Cronbach's alpha was 0.83.

#### Patient Health Questionnaire-9

The PHQ-9 is a self-report rating scale for measuring the severity of the depressive symptoms of an individual (29). Each of the nine items can be rated on a three-point Likert scale ranging from 0 (not at all) to 3 (nearly every day). A higher score reflects a severe degree of depression (0–4 = minimal depression, 5–9 = mild depression, 10–14 = moderate depression, 15–19 = moderately severe depression, and ≥20 = severe depression). In this study, we applied the Korean version of PHQ-9 scale (www.phqscreeners.com), and a score of 10 was defined as clinical depression. Cronbach's alpha of these items was 0.85.

### Statistical Analysis

RStudio and Microsoft Office Excel 2019 were utilized for the data management and analyses. Descriptive statistics (percentages, mean, and standard deviation) were used to assess the distribution of responses. Skewness and kurtosis were calculated to assess the normality assumption. The psychometric properties of the SAVE-6 for patients with cancer were assessed utilizing both classical and modern test theory approaches. Under the classical test theory approach, a confirmatory factor analysis (CFA) was run to test the factor structure of the SAVE-6 for cancer patients. The CFA model fits were –χ^2^/df ratio, comparative fit index (CFI), Tucker-Lewis index (TLI), root-mean-square-error of approximation (RMSEA), and standardized root-mean-square residual (SRMR) values. Multi-group CFA with configural invariance testing was conducted to examine whether the SAVE-6 can measure the anxiety response in the same way across factors such as sex, having depression (PHQ-9 ≥ 10), being in a state of complete remission, or currently undergoing cancer treatment. These results were confirmed whether the CFA with metric and scale constraints are sufficiently corroborated by the model fit. An item analysis was run to estimate the corrected item-total correlation and internal consistency reliability [Cronbach's alpha, McDonald's omega, and Split-half reliability (odd-even)]. Additionally, the average inter-item correlation, standard error of measurement, and Ferguson's delta were calculated.

Under the modern test theory approach, assumptions [unidimensionality (Loevinger's H coefficient), local dependence (*p*-values of G^2^), and monotonicity (number of significant violations and Crit value)] were estimated. Subsequently, a graded response model, a modern test theory model for polytomous items, was run to estimate the discrimination/slope parameters and difficulty/threshold parameters of the SAVE-6 for cancer patients. In addition, the IRT reliability and Rho coefficient were calculated.

Subsequently, Pearson product-moment correlation was run to estimate the correlation between SAVE-6, CAS, and PHQ-9. Two-independent sample *t*-tests were run to assess the mean differences in the SAVE-6 scores between individuals having depression (PHQ-9 ≥ 10) vs. no depression (PHQ-9 < 10).

## Results

### Demographic Characteristics

Among a total of 558 participants, 281 (50.4%) participants were men (Table 1) and 164 (29.4%) had occupations. Their mean age was 59.6 ± 11.7 years. Half of the participants were diagnosed with solid tumor (*N* = 279, 50.0%), and 36.7% of the participants were diagnosed with leukemia (*N* = 205, 36.7%). A total of 402 participants (72.0%) were undergoing cancer treatment, and 106 (19.0%) were in a state of complete remission.

**Table 1:** Demographic and clinical characteristics of the study subjects (*n* = 558).

	***N*** **(%)**,
**Variable**	**Mean ±SD**
Sex (Male), *N* (%)	281 (50.4%)
Age (years)	59.6 ± 11.7
Cohabitants, presence, *N* (%)	478 (85.7%)
Education level, graduate degree, *N* (%)	121 (21.7%)
Religion, presence, *N* (%)	363 (65.1%)
Occupation, presence, *N* (%)	164 (29.4%)
Current alcohol use, presence	49 (8.8%)
Current tobacco use, presence	26 (4.7%)
Psychiatric illness, presence	47 (8.4%)
**Cancer types**	
Solid tumor	279 (50.0%)
Leukemia	205 (36.7%)
Others	74 (13.2%)
**Cancer stages (TNM classification**, ***N*** **=** 449^*^)	
Stage I, II, III	279 (62.1%)
Stage IV	170 (37.9%)
Current cancer treatment, presence	402 (72.0%)
Complete remission, yes	106 (19.0%)
**Questionnaires, score**	
Stress and anxiety to viral epidemics-6	13.7 ± 4.7
Corona anxiety scale	1.0 ± 2.2
Patient health questionnaire-9	3.7 ± 4.6

* *Patients with cancer types excluding hematologic and liver cancer*.

*SD, standard deviation*.

### CFA

**Figure 1:**
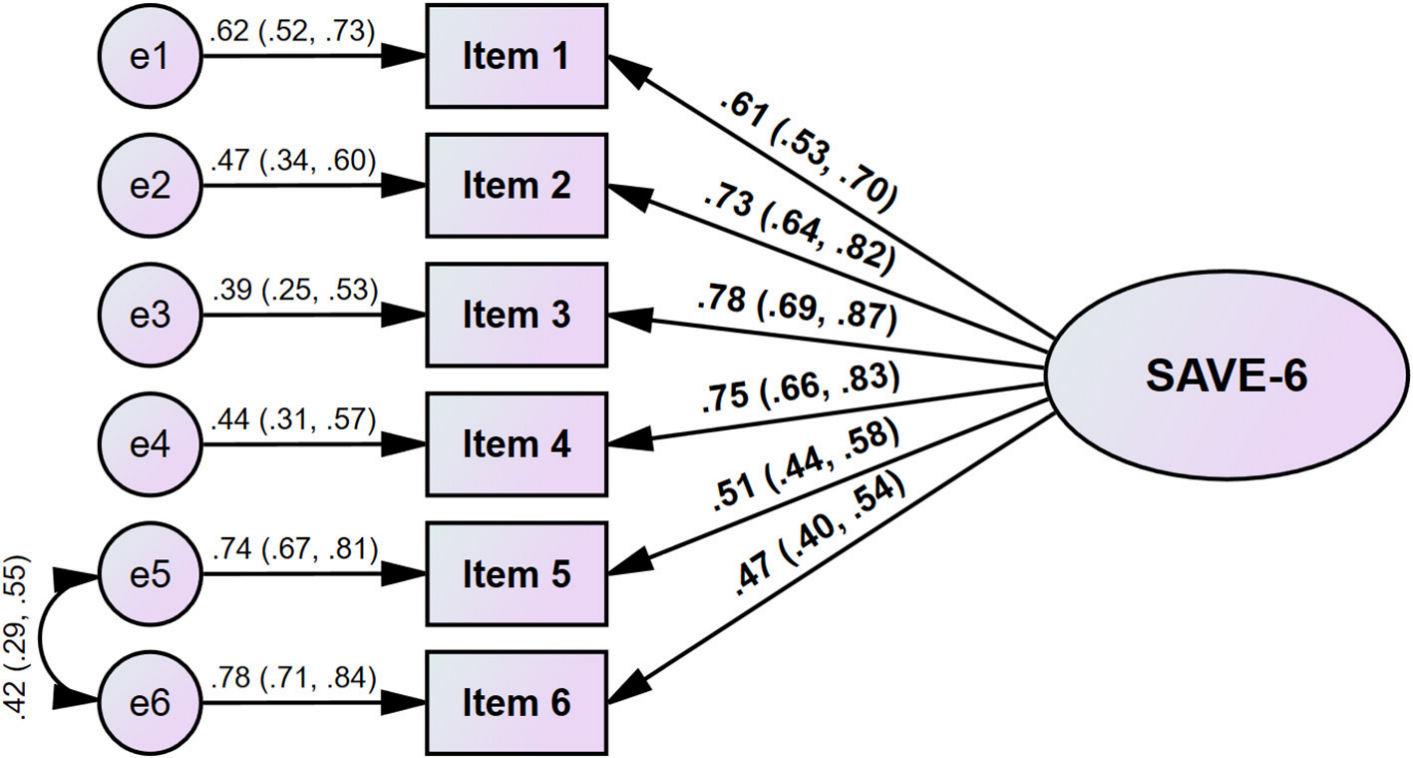
Factor structure of the SAVE-6 among cancer patients.

Initially, the CFA results showed that the model fits of the single factor structure of the SAVE-6 for the cancer patients (χ^2^/df = 6.476, CFI = 0.958, TLI = 0.930, RMSEA = 0.099, and SRMR = 0.085) were not satisfactory. The modification indices were examined and found that a higher covariance was found between items 5 and 6. The model was revised, and the revised model was re-run. This revised model (Figure 1) had a good model fit (χ^2^/df = 62.773, CFI = 0.988, TLI = 0.977, RMSEA = 0.059, and SRMR = 0.057). The factor loadings ranged between 0.470 (0.397, 0.543) and 0.783 (0.693, 0.871) (Table 2; Figure 1). The multi-group CFA with configural invariance testing showed that the SAVE-6 can measure the anxiety response in the same way across different variables, including sex (CFI = 0.966, TLI = 0.944, RMSEA = 0.091, RSMR = 0.082), having clinical depression (PHQ-9 ≥ 10, CFI = 0.964, TLI = 0.939, RMSEA = 0.092, RSMR = 0.084), being in a state of complete remission (CFI = 0.964, TLI = 0.940, RMSEA = 0.093, RSMR = 0.085), or currently undergoing cancer treatment (CFI = 0.963, TLI = 0.939, RMSEA = 0.094, RSMR = 0.086). Multigroup CFA with metric or scale invariant model also showed similar results (Supplementary Table 1).

**Table 2:** Item properties of the SAVE-6 scale among cancer patients.

**Items**	**Response scale (%)**					**Descriptive**				**CITC**	**CID**	**Factor loading (95% CI)**
	**0**	**1**	**2**	**3**	**4**	**M**	**SD**	**Skewness**	**Kurtosis**			
Item 1	2.5	7.7	15.6	53.6	20.6	2.82	0.93	−0.97	0.91	0.529	0.802	0.614 (0.528, 0.700)
Item 2	4.7	10.8	19.2	49.5	15.9	2.61	1.03	−0.81	0.21	0.628	0.782	0.630 (0.641, 0.819)
Item 3	7.3	15.6	22.4	43.5	11.1	2.36	1.10	−0.57	−0.46	0.663	0.773	0.782 (0.693, 0.871)
Item 4	8.8	15.8	24.9	43.9	6.6	2.24	1.08	−0.59	−0.48	0.639	0.778	0.748 (0.663, 0.833)
Item 5	20.3	35.7	18.8	21.1	4.1	1.53	1.15	0.35	−0.91	0.546	0.799	0.507 (0.437, 0.578)
Item 6	11.8	19.4	19.4	41.6	7.9	2.14	1.17	−0.43	−0.90	0.514	0.807	0.470 (0.397, 0.543)

*0, never; 1, rarely; 2, sometimes; 4, often; 5, always; M, mean; SD, standard deviation; CITC, corrected item-total correlation; CID, Cronbach's alpha, if item deleted; CI, confidence interval*.

### Graded Response Model Analysis

The result regarding unidimensionality (Loevinger's H coefficient = 0.485; Table 3) suggested that the SAVE-6-for cancer patients is moderately unidimensional. The *p*-values of G^2^ (Supplementary Table 2) are more than 0.05 and suggest the absence of local dependence between the items. Additionally, Supplementary Table 2 showed that there are no significant violations and Crit values were also below 40 (recommended cut off < 40) (30). These suggest the absence of monotonicity of the items. Overall, the modern test theory assumptions were met. Supplementary Table 3 shows that the discrimination/ slope parameters (α) are ranged between 1.360 and 2.654 (mean = 2.043). Items 5 and 6 had a high slope, and the remaining items had a very high slope. These items were found to be good in discriminating among cancer patients in anxiety and stress assessed by SAVE-6. Supplementary Table 3 also shows that a lower latent trait is required to endorse all the items, except item 5. In these items, only b_4_ coefficients are positive and the rest of the coefficients are negative. Item 5 is the most difficult item for assessing the anxiety among cancer patients. The scale information curve (Supplementary Figure 1) shows that this scale provides additional information about the individuals between −1.75 and −0.25, θ levels. There are two peaks in the curve; additionally, these might be due to the polytomous nature of the data.

**Table 3:** Scale-level psychometric properties of the SAVE-6 among cancer patients.

**Psychometric properties**	**Scores**	**Suggested cut off**
Floor effect	1.1	15%
Ceiling effect	1.4	15%
Mean inter-item correlation	0.434	Between 0.15 and 0.50
Cronbach's alpha	0.819	≥0.7
McDonald's Omega	0.818	≥0.7
Split-half reliability (odd-even)	0.881	≥0.7
Standard error of measurement	1.99	Smaller than SD (2.34)/2
Ferguson delta	0.972	≥0.9
Loevinger's *H* coefficients	0.485	–
*Rho* coefficient	0.823	≥0.7
IRT reliability	0.856	≥0.7
**Model fits of confirmatory factor analysis**		
χ^2^ (df, *p*-value), χ^2^/df	22.181 (8, 0.005), 2.773	Non-significant, <5
CFI	0.988	>0.95
TLI	0.977	>0.95
RMSEA (90% CI value) (*p*-value)	0.059 (0.029, 0.085) (0.312)	<0.08
SRMR	0.057	<0.08

### Reliability and Evidence Based on Relations to Other Variables

The SAVE-6 scale showed good reliability [Cronbach's alpha = 0.819, McDonald's Omega = 0.818, Split-half reliability (odd-even) = 0.881, Table 3]. A Cronbach's alpha was measured as 0.773–0.807 if an item was dropped. The mean inter-item correlation (.434) was between the recommended range (0.15–0.50). The SAVE-6 showed good IRT reliability (0.856) and Rho coefficient (0.823). It also had good discrimination power (Ferguson's delta = 0.972). The SAVE-6 total score was significantly correlated with the CAS [r = 0.348 (95% CI, 0.273–0.419), *p* < 0.001] and PHQ-9 score [r = 0.251 (95% CI, 0.172–0.328), *p* < 0.001]. The SAVE-6 score was significantly high among patients with cancer who had depression [PHQ-9 ≥ 10, *t*_(556)_ = 3.197, *p* < 0.001].

## Discussion

In this study, we aimed to examine the psychometric properties of SAVE-6 among patients with cancer in stressful situations during the COVID-19 pandemic, and to explore whether the SAVE-6 scale can measure their anxiety response specifically to the viral epidemic. The results of the current study confirmed that the SAVE-6 showed good validity and reliability when applied to patients with cancer, similar to the previous studies applied among the general population (20, 23, 24). Furthermore, we observed that the SAVE-6 could measure anxiety responses similarly across factors, such as sex, having clinical depression, being in state of complete remission, or currently undergoing cancer treatment.

The SAVE-6 scale was developed for measuring the anxiety response to the viral epidemic, and it was validated among the general population. Patients with cancer have experienced psychological distress during the COVID-19 pandemic (31), and the assessment and management of their psychological distress specific to the pandemic itself is important. We have tried to examine the applicability of the SAVE-6 scale for patients with cancer in other samples (32); however, the psychometric properties could not be fully explored. The current study was conducted to confirm whether the construct validity or reliability of the SAVE-6 is also good among patients with cancer.

In CFA, the first model fits were not satisfactory; additionally, we checked the modification indices and observed that an error variance of items 5 and 6 highly correlated. It reflected that item 5 (Are you worried that others might avoid you even after the infection risk has been minimized?) and item 6 (Do you worry that your family or friends may become infected because of you?) might be connected. The proportion of responses of “never” or “rarely” to item 5 (56%) and item 6 (31.2%) is too high compared to those of other items (Table 2). These two items are not questions regarding the physical condition of the patients but are related to other people and the patient's infectivity; furthermore, infectivity can be risky to other individuals. Although we cannot directly compare the differences in the responses between patients with cancer and normal controls in this study, we can speculate that patients with cancer may focus on the symptoms or their own infectivity rather than other individuals from the results of other items (1, 2, 3, and 4) that were related to infectivity to themselves. However, the revised model had good model fits; the factor loading of item 6 was below 0.50 (0.47), when a value of over 0.60 was generally considered as an acceptable factor loading value. Nevertheless, a value of 0.5 of factor loading may also be accepted if the reliability is over 0.60 (33). We observed a good reliability of the SAVE-6 (Cronbach's alpha of 0.819 and McDonald's Omega of 0.818), and accepted item 6 to be included in the final single-structure model of the SAVE-6, when it was applied to patients with cancer.

This study had several limitations. First, it was conducted among patients with cancer who visited the hospital and agreed to participate. Patients with cancer worry about being infected during the COVID-19 pandemic, and the differences in the characteristics or psychiatric symptoms between patients who visited the hospital and those who did not visit might have influenced the results. Second, we could not gather information on COVID-19, such as “experience of being infected” or “quarantined.” The results might have been influenced by the proportion of patients who experienced being infected or quarantined. Although a precise prediction cannot be made, we can speculate that the mean level of virus-related anxiety may increase, as more participants become infected. Finally, this study was conducted among patients with cancer in one hospital. Further study will need to be conducted in multiple centers to explore whether the reliability of the SAVE-6 among patients with cancer is maintained or not.

Despite these limitations, we observed that the SAVE-6 is a reliable and valid scale for assessing the anxiety response of patients with cancer during the COVID-19 pandemic. Although the SAVE-6 was developed for assessing the anxiety response of the general population, the results of this study confirmed that it can be used with reliability and validity among patients with cancer.

## Data Availability Statement

The raw data supporting the conclusions of this article will be made available by the authors, without undue reservation.

## Ethics Statement

The study protocol was approved by the Institutional Review Board of the Ulsan University Hospital (2020-1055). The patients/participants provided their written informed consent to participate in this study.

## Author Contributions

SC and S-JK: conceptualization, investigations, and resources. HyK, HL, and S-JK: data curation. SC, OA, and HaK: formal analysis. SC, OA, and HyK: methodology. HaK and SC: project administration. EC, HaK, and HL: visualization. HyK, HL, SC, EC, and OA: writing—original draft. All authors: writing—review and editing. All authors contributed to the article and approved the submitted version.

## Conflict of Interest

The authors declare that the research was conducted in the absence of any commercial or financial relationships that could be construed as a potential conflict of interest.

## Publisher's Note

All claims expressed in this article are solely those of the authors and do not necessarily represent those of their affiliated organizations, or those of the publisher, the editors and the reviewers. Any product that may be evaluated in this article, or claim that may be made by its manufacturer, is not guaranteed or endorsed by the publisher.
