# Supplementary material for: The Validation Study of the Stress and Anxiety to Viral Epidemics−6 Scale Among Patients With Cancer in the COVID-19 Pandemic
Source: Front Psychiatry. 2022 Apr 14;13:811083. doi: 10.3389/fpsyt.2022.811083 (PMC9046784; doi:10.3389/fpsyt.2022.811083)

Supplementary Material

**Supplementary Table 1. Measurement invariance**

| **Model** | **χ^2^** | **Df** | **Δ χ^2^** | **Δdf** | **p** | **CFI** | **ΔCFI** | **RMSEA** | **ΔRMSEA** |
| --- | --- | --- | --- | --- | --- | --- | --- | --- | --- |
| **Sex (male vs. female)** | | | | | | | | | |
| Configural | 23.836 | 16 |  |  |  | 0.994 |  | 0.042 |  |
| Metric | 36.220 | 21 | 12.384 | 5 | 0.030 | 0.988 | 0.006 | 0.051 | 0.009 |
| Scalar | 65.332 | 26 | 29.112 | 5 | <.001 | 0.968 | 0.020 | 0.074 | 0.023 |
| **Having depression (PHQ-9 ≥ 10 vs. PHQ-9 <10)** | | | | | | | | | |
| Configural | 24.219 | 16 |  |  |  | 0.993 |  | 0.043 |  |
| Metric | 37.679 | 21 | 13.46 | 5 | 0.019 | 0.986 | 0.007 | 0.053 | 0.010 |
| Scalar | 128.500 | 26 | 90.821 | 5 | <.001 | 0.911 | 0.075 | 0.119 | 0.066 |
| **State of complete remission (yes vs. no)** | | | | | | | | | |
| Configural | 25.023 | 16 |  |  |  | 0.993 |  | 0.045 |  |
| Metric | 32.794 | 21 | 7.771 | 5 | 0.169 | 0.990 | 0.003 | 0.045 | 0 |
| Scalar | 164.856 | 26 | 132.062 | 5 | <.001 | 0.885 | 0.105 | 0.139 | 0.094 |
| **Current undergoing cancer treatment (yes vs. no)** | | | | | | | | | |
| Configural | 25.258 | 16 |  |  |  | 0.992 |  | 0.046 |  |
| Metric | 27.803 | 21 | 2.545 | 5 | 0.770 | 0.994 | -0.002 | 0.034 | -0.012 |
| Scalar | 34.712 | 26 | 6.909 | 5 | 0.227 | 0.993 | 0.001 | 0.035 | 0.001 |

**Supplementary Table 2. Loevinger’s H coefficient, monotonicity, and G^2^ p values of the**

**items**

|  | ***H* coefficients** | **Monotonicity** | | | | **Local dependence G^2^ p values** | | | | |
| --- | --- | --- | --- | --- | --- | --- | --- | --- | --- | --- |
|  |  | **#ac** | **#vi** | **#zsig** | ***Crit*** | **Item 1** | **Item 2** | **Item 3** | **Item 4** | **Item 5** |
| **Item 1** | .71 | 68 | 0 | 0 | 0 |  |  |  |  |  |
| **Item 2** | .71 | 71 | 2 | 0 | 17 | .274 |  |  |  |  |
| **Item 3** | .60 | 99 | 5 | 0 | 14 | .274 | .274 |  |  |  |
| **Item 4** | .58 | 69 | 3 | 0 | 10 | .274 | .274 | .274 |  |  |
| **Item 5** | .52 | 74 | 3 | 0 | 10 | .274 | .274 | .274 | .274 |  |
| **Item 6** | .49 | 84 | 1 | 0 | 11 | .274 | .274 | .274 | .274 | .289 |
| ac = active comparison, vi = violation, zsig = significant violation  Notes: p-values adjusted for false discovery rate (FDR) | | | | | | | | | | |

**Supplementary Table 3. Item fit, slope, and threshold parameters**

| **Items** | **Slope parameter (a)** | **Threshold parameter (b)** | | | |
| --- | --- | --- | --- | --- | --- |
|  |  | **b_1_** | **b_2_** | **b_3_** | **b_4_** |
| **Item 1** | 1.845 | -2.734 | -1.667 | -.832 | 1.105 |
| **Item 2** | 2.555 | -2.008 | -1.170 | -.456 | 1.177 |
| **Item 3** | 2.654 | -1.719 | -.875 | -.170 | 1.438 |
| **Item 4** | 2.251 | -1.710 | -.858 | -.029 | 1.911 |
| **Item 5** | 1.393 | -1.303 | .172 | .957 | 2.834 |
| **Item 6** | 1.360 | -1.892 | -797 | -.029 | 2.283 |
| Notes: p-values adjusted for false discovery rate (FDR) | | | | | |

**Supplementary Figure 1.** **Scale information curve of the SAVE-6 among cancer patients**


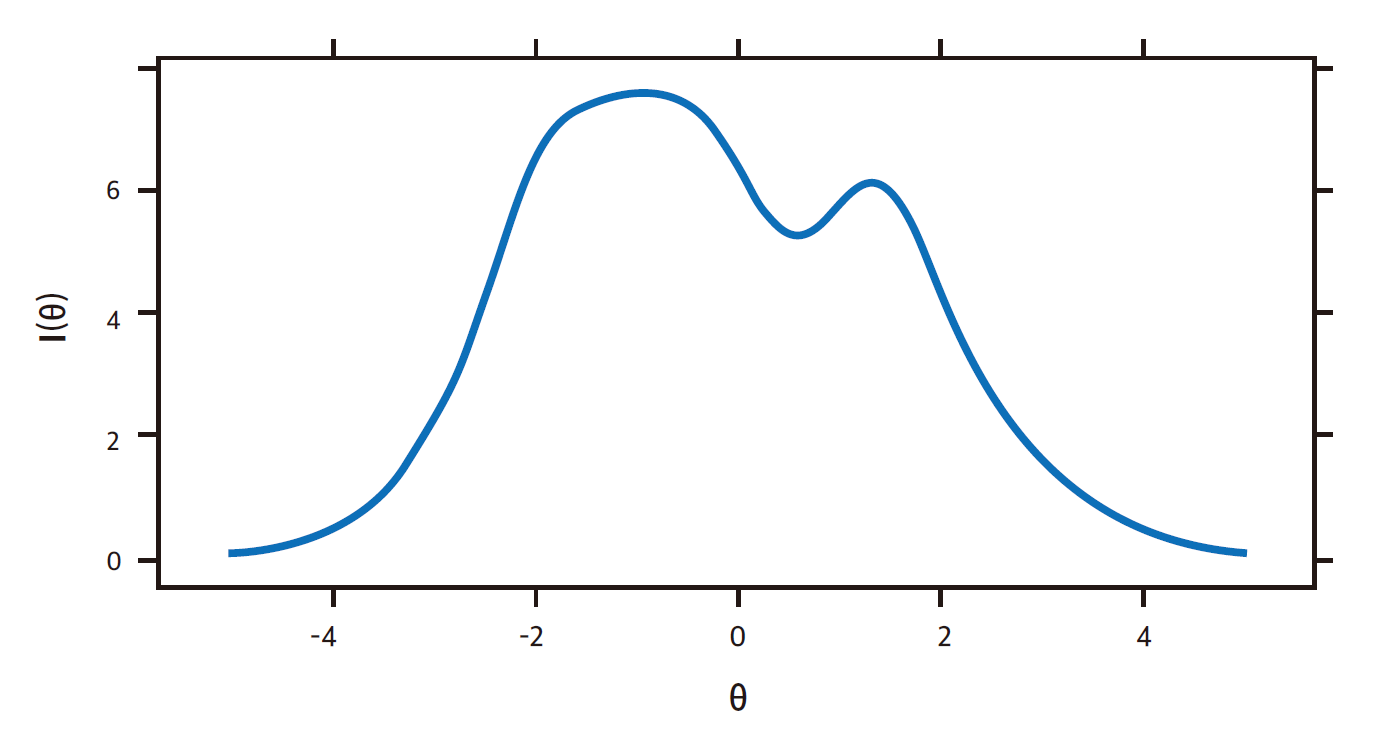

Supplement: Supplementary file 1 [file Data_Sheet_1.docx]
